# Supplementary material for: Patterns of Intron Gain and Loss in Fungi
Source: PLoS Biol. 2004 Nov 30;2(12):e422. doi: 10.1371/journal.pbio.0020422 (PMC532390; doi:10.1371/journal.pbio.0020422)
Supplement: Table S1 — Also available at http://genes.mit.edu/NielsenEtAl/. (4.3 MB ZIP). [file pbio.0020422.st001.zip › NielsenEtAl/html/1301.html]

AN6139.1.NCU00168.1.MG11040.1.FG06642.1


```
 CLUSTAL W (1.82) Multiple Sequence Alignments - Introns Inserted


Sequence 1: NCU00168.1	305 aa
Sequence 2: MG11040.1	303 aa
Sequence 3: FG06642.1	303 aa
Sequence 4: AN6139.1	291 aa
Alignment Length: 307 aa
Number Identitical Residues: 124 aa
Alignment Score (without introns) 6192


MG11040.1 	---MLEYLYYFAGGYTSLVIGLYMMSFAIPKAAFVARILASYICLLVAASFGVVCSLILR
NCU00168.1	MASLLYYPVYFLEGCVALTIGFYMLSLILPRAAFVARSLASYLSLLLCAGFGVITSIAFS
FG06642.1 	-MSISSVLTYFALGYAALTIFLYAMSLVVPQAAFAARGLASYVSLLVVAAYGVFASIFLT
AN6139.1  	-MSLLYYIASGASTYIAFTASLFLVGQKVPRASFVARCLASYGSLLVCAMYGVVASIVLR
          	  ::            ::.  :: :.  :*:*:*.** **** .**: * :**. *: : 

MG11040.1 	FFGAEQNAQHYTARVFMWLMLAATGVTFEIIDPKGHLSKEENRPAVLVGNHQTELDVLML
NCU00168.1	LLGHGQSAQWAVGRSFKYVMALTTGVRFKIDDPKNILGK--TRPAVFIGNHQTELDVLML
FG06642.1 	LIGKQGIAQWAVGRSFHYLMLYTTGVEFVIEDPDNVLGT--TRPAVFVGNHQTELDVLML
AN6139.1  	VVGYGRISQWATARSFKWVMRFTTGVRFDIVEGKEYLST---RPAVIIGNHQSELDVLML
          	..*    :*  ..* * ::*  :*** * * : .  *..   ****::****:*******

MG11040.1 	GCLFPEHCSVTAKASLRRTPFLGWFMSLSGTIFIDRKNSADARQAMSGAVNQIRTKRQTV
NCU00168.1	GCMFPKYCSVTAKSSLRKTPFLGWFMSLSGSIFIDRASKTDARAAMAGAANQIQKKQQSV
FG06642.1 	GRMFPKYCSVTAKASLKKVPFLGWFMSLSGSIFIDRKNSKDARDAMKGAASEIQSKRQSV
AN6139.1  	GEIFPPYCSVTAKKSLRYVPFLGWFMALSRTVFIDRANRQTAVKAFDSAAEEMRSHRQSV
          	* :** :****** **: .*******:** ::**** .   *  *: .*..:::.::*:*

MG11040.1 	YMFPEGTRSYTREPILLPFKKGAFHLAVQAQVPIVPCVVANYSHVLWIKGLVFNAGKIPV
NCU00168.1	YMFPEGTRSYSKEPELLPFKKGAFHLAVQAQVPIVPVVVANYSHVLNVKDFVFNAGVIPM
FG06642.1 	YMFPEGTRSYAKEPMLLPFKKGAFHLAVEAGVPIVPCVVANYSHVLYPKTFTFNSGKIPV
AN6139.1  	FIFAEGTRSYSEKPELLPFKKGAFHLAVKAGVPIVPVVVENYSHILAPKKFRFEAGSIKV
          	::*.******:.:* *************:* ***** ** ****:*  * : *::* * :

MG11040.1 	KV1LDPIPTTGMTAADVDGLCKKTYDLMHQELITLTSKARGEPVT-VPSSMNPTGIIEAT
NCU00168.1	KV1LDPIDTTGLTTADVDELCKNTRDLMLKELIALTAKARGQSVSDIPVANKYPSVAKAS
FG06642.1 	KV1LKPIPTKGLTAADVEELTRTTRELMLNELVTVTAKARGQPMAVPASLSDSTGKSSAI
AN6139.1  	KV1LPPISTDGLTAADVDGLTTSTRESMLNTLLELSNAG----PADLPSSSK--GQSTAV
          	** * ** * *:*:***: *  .* : * : *: ::  .     :  .   .  .   * 

MG11040.1 	NGKPKHSS
NCU00168.1	GTDMQVAA
FG06642.1 	DTNGSAQ-
AN6139.1  	DL------
          	.
```
